# Comprehensive bioinformatics analysis reveals the role of cuproptosis-related gene Ube2d3 in myocardial infarction

**DOI:** 10.3389/fimmu.2024.1353111

**Published:** 2024-02-19

**Authors:** Ming Yang, Yucheng Wang, Liming He, Xinxin Shi, Shuwei Huang

**Affiliations:** ^1^ The First Clinical Medical College, Zhejiang Chinese Medical University, Hangzhou, China; ^2^ Department of Cardiology, the First Affiliated Hospital of Zhejiang Chinese Medical University, Zhejiang Provincial Hospital of Chinese Medicine, Hangzhou, China

**Keywords:** UBE2D3, cuproptosis, bioinformatics, cardiomyocyte myocardial infarction, cardiomyocyte

## Abstract

**Background:**

Myocardial infarction (MI) caused by severe coronary artery disease has high incidence and mortality rates, making its prevention and treatment a central and challenging aspect of clinical work for cardiovascular practitioners. Recently, researchers have turned their attention to a novel mechanism of cell death caused by Cu^2+^, cuproptosis.

**Methods:**

This study integrated data from three MI-related bulk datasets downloaded from the Gene Expression Omnibus (GEO) database, and identified 16 differentially expressed genes (DEGs) related to cuproptosis by taking intersection of the 6378 DEGs obtained by differential analysis with 49 cuproptosis-related genes. Four hub genes, Dbt, Dlat, Ube2d1 and Ube2d3, were screened out through random forest analysis and Lasso analysis. In the disease group, Dbt, Dlat, and Ube2d1 showed low expression, while Ube2d3 exhibited high expression.

**Results:**

Focusing on Ube2d3 for subsequent functional studies, we confirmed its high expression in the MI group through qRT-PCR and Western Blot detection after successful construction of a MI mouse model by left anterior descending (LAD) coronary artery ligation, and further clarified the correlation of cuproptosis with MI development by detecting the levels of cuproptosis-related proteins. Moreover, through *in vitro* experiments, Ube2d3 was confirmed to be highly expressed in oxygen-glucose deprivation (OGD)-treated cardiomyocytes AC16. In order to further clarify the role of Ube2d3, we knocked down Ube2d3 expression in OGD-treated AC16 cells, and confirmed Ube2d3’s promoting role in the hypoxia damage of AC16 cells by inducing cuproptosis, as evidenced by the detection of MTT, TUNEL, LDH release and cuproptosis-related proteins.

**Conclusion:**

In summary, our findings indicate that Ube2d3 regulates cuproptosis to affect the progression of MI.

## 1 Introduction

Myocardial infarction (MI) is a cardiovascular disease that poses a significant threat to human health and life (1). It primarily arises from the formation of coronary atherosclerotic thrombosis or an imbalance of myocardial oxygen supply and demand. When atherosclerotic thrombosis ruptures, the released plaque can accumulate platelets, leading to coronary artery occlusion and subsequently myocardial ischemia and necrosis (2), accompanied by severe complications such as arrhythmias, cardiogenic shock, and heart failure. Statistics in recent years indicates a concerning trend in the incidence of MI, with a gradual shift towards affecting younger individuals (3).

While the prognosis of MI has shown improvement in recent years due to advances in early reperfusion strategies, drug therapy, standardized care, and identification of susceptible patient subsets, it remains one of the leading causes of death worldwide (4). Therefore, identifying key causative factors affecting the development of MI remains the focus of clinicians. Cuproptosis, as a novel mode of cell death distinct from other programmed cell death such as apoptosis, pyroptosis, pyrosis, necrosis, and ferroptosis (5), may be a crucial determinant affecting the progression of MI (6). Previous investigations have indicated the close involvement of cuproptosis in diseases such as cancer, Wilson’s disease, and neurodegenerative diseases (7). In this study, our objective is to identify cuproptosis-related genes (CRGs) that affect the development of MI via bioinformatics-based methodologies, seeking to provide a theoretical basis for the clinical discovery of new pharmacodynamic targets.

## 2 Materials and methods

### 2.1 Data acquisition

The Gene Expression Omnibus (GEO) is an international public repository where researchers globally share a wide range of genomic data, including microarray chips, second-generation sequencing, and other forms of high-throughput genomic data, for free download. Therefore, we chose to download MI-related bulk datasets, namely GSE104187, GSE153485, GSE206281, and a single-cell dataset GSE214611, from GEO. There are four samples in the GSE104187 dataset, including two MI samples and two normal samples; ten samples in the GSE153485 dataset, with five MI samples and five normal samples; six samples in the GSE206281 dataset, consisting of three MI samples and three normal samples; seven samples in the GSE214611 dataset, comprising four MI samples and three normal samples. Additionally, we sorted out 49 genes associated with cuproptosis from the published literature (**Supplementary Table 1**) (1, 8, 9).

### 2.2 Data processing

We integrated the three bulk datasets and subsequently carried out batch effect elimination using the SVA package (Version 3.48), following which we conducted data standardization using the limma package (Version 3.56).

### 2.3 Differential expression analysis

In our effort to identify genes influencing MI progression, we performed a differential expression analysis on the integrated dataset using the amplicon package (Version 1.19), and set a criterion of |logFC|>0.2 & adjust. P-value<0.05 for screening differentially expressed genes (DEGs). The results were visually represented through volcano maps using the ggplot2 package (Version 1.16.0).

### 2.4 Functional enrichment analysis

The clusterProfiler package (Version 4.6.2), org.Hs.eg.db package (Version 3.16.0) and ggplot2 package (Version 3.4.2) were used for performing GO and KEGG functional enrichment analysis of the DEGs, with parameters set as follows: p-value Cutoff<0.05, q-value Cutoff<0.05, and pAdjustMethod to Benjamini-Hochberg (BH). Venn diagrams were generated using the ggVennDiagram (Version 1.2). gseKEGG in clusterProfiler (Version 4.8) were applied for performing single-sample gene set enrichment analysis (ssGSEA).

### 2.5 Machine learning algorithms

In our analysis aimed at screening out key genes influencing MI progression, we employed the randomFores package (Version 4.7) for conducting random forest analysis. Setting the seed to 123 and the number of trees to 500, we finally selected the top seven genes based on their importance SD. At the same time, we employed the glmnet package (Version 4.1-7) for lasso analysis, specifying the family as “binomial”. Through binomial machine learning, we obtained six characteristic genes.

### 2.6 Immunoinfiltration analysis

To investigate whether there is a correlation between key genes and immunity and whether immunity plays an important role in the disease, we conducted immunoinfiltration analysis using the CIBERSORT package.

### 2.7 Single-cell analysis

We performed single-cell analysis on the GSE214611 dataset using the Seurat package (Version 4.3.0). Quality control of the single-cell data was conducted, and cells meeting the following criteria were retained: (1) cells with a gene count ranging from 200 to 5000; (2) cells with a mitochondrial ratio below 5%; (3) cells with an RNA count of less than 20,000.

### 2.8 Animals used in experiments

For performing animal experiments, we purchased 12-week-old male C57BL/6 mice from Zhejiang Vital River Laboratory Animal Technology Co., Ltd. and raised them under constant temperature and humidity (23°C, 65%) within a 12/12-hour light/dark regime. All experimental procedures were conducted in strict adherence to relevant provisions outlined in the National Laboratory Animal Welfare Ethics, and this animal study was approved by the Laboratory Animal Welfare Ethics Committee, with approval number of ZJCLA-IACUC-20040153.

### 2.9 Animal model construction

We randomly divided 20 mice into two groups: MI and Sham. Nine mice survived in the MI group, while ten survived in the Sham group. Afterwards, three mice from each group were randomly selected for TTC staining, and six for related molecular experiments. In the MI group, mice underwent endotracheal intubation, followed by ligation of the LAD coronary artery to induce MI. After inducing anesthesia until the muscle strength decreased to level 0, the mice were taken out and fixed on a sticky mouse board. Anesthesia was maintained by pressing the nasal cavity against the mask. A small incision, approximately 1.5cm above the heart, was made in the left chest 3-4 rib space using small scissors. Using a 6-0 silk thread, the anterior descending branch was ligated at 2 mm below the left atrial appendage, tightening the infarct blood vessel and muscle. The needle insertion depth was about 1.5mm. The left auricle displayed purplish-red congestion, and the myocardium gradually changed from bright red to pale after vasculature, with gradual weakening of contraction. By connecting the limb leads of the small animal electrocardiogram detection system, if the electrocardiogram revealed an upward arching of the ST segment, accompanied by the appearance of J waves after the ST segment, the successful establishment of the MI model was indicated. After turning off the anesthetic mask, the skin on both sides was aligned using 3-0 silk for pouch sutures to close the chest. The incision was disinfected with alcohol. Mice were positioned prone on a specialized small animal special electric blanket to facilitate awakening and rewarming. In the Sham group, mice underwent an identical procedure, excluding the step of coronary artery ligation.

### 2.10 Cell culture and grouping

Human cardiomyocytes AC16 (Pricella, CL-0790) were cultured in DMEM/F12 medium containing 10% fetal bovine serum at 37°C in an incubator with 5% CO_2_. To create the cell model, we exposed the cells to oxygen-glucose deprivation (OGD), during which they were cultured in sugar-free DMEM/F12 medium in a hypoxic incubator at 37°C with 94% N_2_, 1% O_2_, and 5% CO_2_ for 24 h. Cells in the control group were cultured under standard conditions.

### 2.11 TTC staining

The collected mouse heart tissue sections were immersed into 2% TTC solution in a light-protected environment, and incubated in a 37°C incubator for 30 min. To ensure complete staining, the container was gently agitated every five minutes. Subsequently, images were captured to observe the heart tissue infarction area.

### 2.12 Hematoxylin-eosin staining

The heart tissue sections were subjected to HE staining using relevant staining kit (Beyotime, C0105M) to visualize and analyze the morphological characteristics of the heart tissue.

### 2.13 DLAT oligomerization

We used DLAT Rabbit pAb (ABclonal, A14530) to detect Dlat oligomerization in the mouse heart tissue or cardiomyocytes.

### 2.14 qRT-PCR

We extracted RNA from mouse heart tissue and cardiomyocytes using the Trizol reagent (Beyotime, R0016). Subsequently, cDNA synthesis was carried out using the SuperScript™ VILO™ cDNA Synthesis Kit (Invitrogen, 11754050). The quantitative PCR procedure was executed according to the instructions provided by the BeyoFast™ SYBR Green qPCR Mix (Beyotime, D7501). The primers used in this experiment are mmu-Ube2d3-F: CACGAGAACAGTTGCCCAAGATTC; mmu-Ube2d3-R: CAGCATGTTAGAGCAGTGAGGATTG; hsa-Ube2d3-F: GGGCATTACCATTCTTCACCTTTCC; hsa-Ube2d3-R: GTGGAAGTGGAGGAGTCACAGATG; mmu-GAPDH-F: GGCAAATTCAACGGCACAGTCAAG; mmu-GAPDH-R: TCGCTCCTGGAAGATGGTGATGG; hsa-GAPDH-F: GATCATCAGCAATGCCTCCT; hsa-GAPDH-R: TGTGGTCATGAGTCCTTCCA.

### 2.15 Western blot

We extracted the total protein from mouse heart tissue and cardiomyocytes with a lysis buffer containing 1% protease inhibitor. Protein quantification was carried out using the BCA method (Beyotime, #P0012S), followed by subsequent procedures including electrophoresis, membrane transfer, antibody incubation, and color development. The primary antibodies included Ube2d3 (Invitrogen, PA5-119881), FDX1 (Abcam, ab108257), FDX1 (Invitrogen, PA5-59653), SLC31A1 (Invitrogen, PA1-16586), Dlat (ABclonal Technology, A8814), Dlat (Invitrogen, PA5-59298) and Lipoic acid (abcam, ab58724). GAPDH (Invitrogen, PA1-988) was selected as the internal reference protein.

### 2.16 Immunofluorescence

To assess neutrophil infiltration in the mouse heart tissue, we used the Anti-Neutrophil antibody (abcam, ab53457) for immunofluorescence analysis.

### 2.17 ELISA

The expression of respective inflammatory factors in the mouse heart tissue was assessed using ELISA assay kits for IL-6 (Beyotime, PI326), IL-1β (Beyotime, PI301), TNF-α (Beyotime, PT512), and MCP-1 (Beyotime, PC125).

### 2.18 MTT assay

Cell viability was assessed following the instructions provided by the MTT kit (Beyotime, C0009S).

### 2.19 Detection of hypoxic damage of cells

Cardiomyocyte damage was assessed following the instructions provided by the LDH Cytotoxicity Assay Kit (Beyotime, C0017). Cell death was evaluated according to the instructions provided by the One Step TUNEL Apoptosis Assay Kit (Beyotime, C1086).

### 2.20 Statistical analysis

Statistical analysis in the bioinformatics section was carried out using the R language. In the experimental part, IBM SPSS Statistics software was used for statistical data analysis, and GraphPad Prism 9 was subsequently used to draw bar charts. Results were expressed as mean ( $\bar{\text{x}}$ ) ± standard deviation (SD), with all measurement data conforming to a normal distribution. Data comparison between groups was performed using t-tests. *P*<0.05 indicates a statistically significant difference.

## 3 Results

### 3.1 Data processing

**Figure 1:**
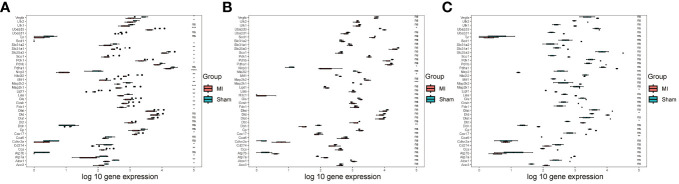
Data processing. **(A)** Box diagram showing the differential analysis results of CRGs in the GSE153485 dataset; **(B)** Box diagram showing the differential analysis results of CRGs in the GSE206281 dataset; **(C)** Box diagram showing the differential analysis results of CRGs in the GSE104187 dataset.

Initially, we performed differential analysis on CRGs in the three downloaded bulk datasets. This analysis revealed significant differences in 26 CRGs in the GSE153485 dataset (**Figure 1A**), five CRGs in the GSE206281 dataset (**Figure 1B**), and four CRGs in the GSE104187 dataset (**Figure 1C**). To investigate DEGs affecting MI development, we merged the three bulk datasets, performed batch effect removal (**Supplementary Figures 1A, B**) and conducted data normalization (**Supplementary Figures 1C, D**).

### 3.2 Differential expression analysis

**Figure 2:**
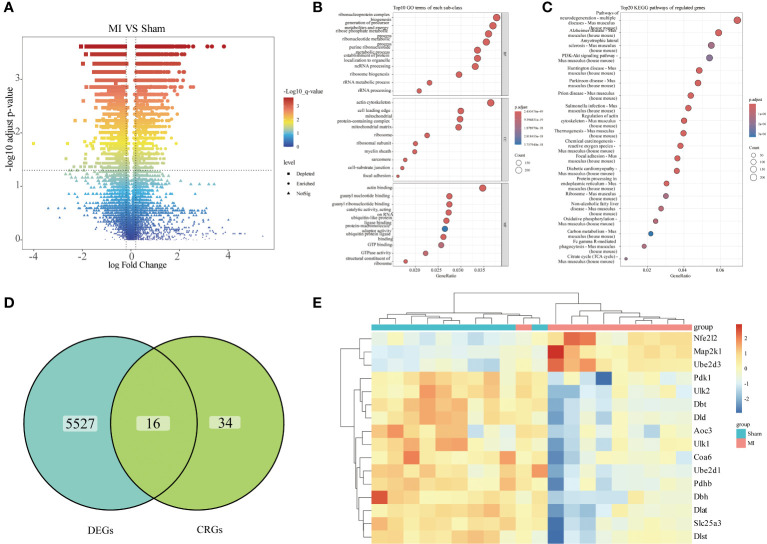
Differential expression analysis. **(A)** Volcano plots illustrating differential analysis results; **(B)** Dot plot presenting GO functional enrichment analysis results of the DEGs (BP: Biological Process, CC: Cellular Components, MF: Molecular Function); **(C)** Dot plot displaying KEGG pathway enrichment analysis results of the DEGs; **(D)** Venn diagram showing the differentially expressed CRGs; **(E)** Heatmap illustrating the expression of differentially expressed CRGs across individual samples.

To identify genes that influence MI progression, we analyzed the differences in the combined dataset, comparing samples from the disease and control groups. A total of 5543 DEGs were identified, comprising 3097 down-regulated and 2446 up-regulated ones (**Figure 2A**). Subsequent functional enrichment analysis categorized these DEGs based on their functions, revealing their primary enrichment in ribonucleoprotein complex, actin cytoskeleton, actin binding and PI3K-AKT signaling pathway (**Figures 2B, C**). Given the focus on cuproptosis in this study, we aimed to screen out genes associated with cuproptosis that might influence MI progression. To achieve this goal, we plotted a Venn diagram illustrating the overlap of DEGs with CRGs, resulting in the identification of 16 cuproptosis-related DEGs (**Figure 2D**), and a heatmap was subsequently generated to visualize their gene expression patterns in each group (**Figure 2E**).

### 3.3 Hub gene screening

**Figure 3:**
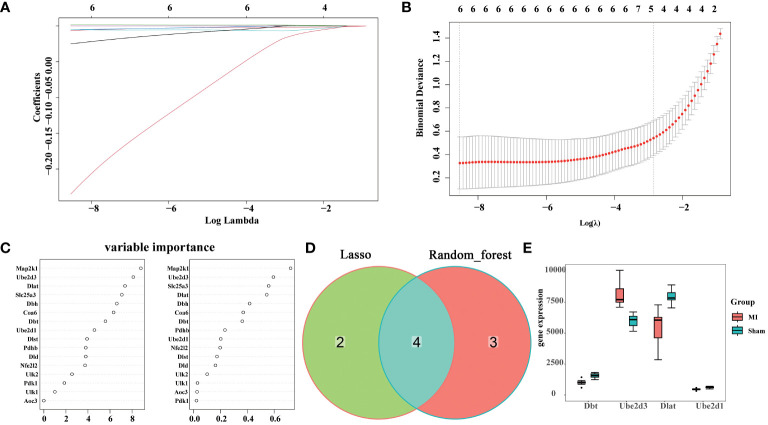
Hub gene screening. **(A)** Coefficient distribution plots displaying the coefficients for each gene; **(B)** Diagram of parameters; **(C)** Variable importance of genes; **(D)** Venn diagram illustrating the identification of hub genes through the integration of two machine learning algorithms; **(E)** Boxplots showing the expression of the identified hub genes across different experimental groups.

To further identify key cuproptosis-related DEGs that affect MI, two different machine learning algorithms were applied: LASSO algorithm and random forest algorithm. Through lasso analysis, we screened out six characteristic genes (**Figures 3A, B**), while through random forest analysis, we identified 16. To narrow down the selection of key genes, we selected the top seven genes with the largest importance SD value for subsequent analysis (**Figure 3C**). Then, we obtained four hub genes, Dlat, Ube2d1, Ube2d3, and Dbt, by taking intersection of the results of the two machine learning analyses (**Figure 3D**), and plotted their expression boxplots (**Figure 3E**). To understand the pathways regulated by these four key genes, ssGSEA analysis was conducted. The results revealed that Dlat (**Supplementary Figure 2A**), Ube2d1 (**Supplementary Figure 2B**), Ube2d3 (**Supplementary Figure 2C**), and Dbt (**Supplementary Figure 2D**) may all be involved in Citrate cycle (TCA cycle). This outcome suggests their potential involvement in cuproptosis.

### 3.4 Immunoinfiltration analysis

**Figure 4:**
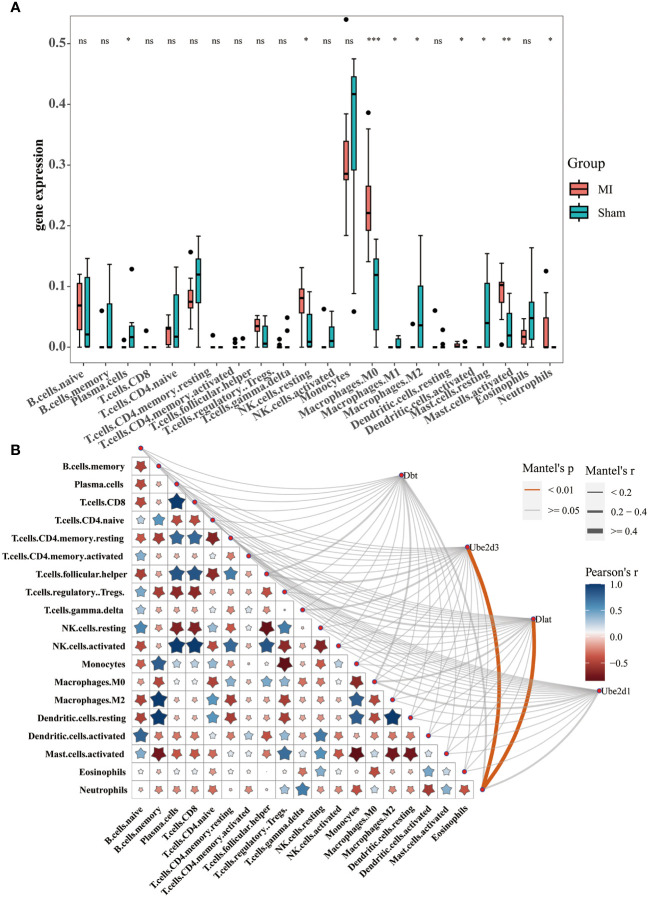
Immunoinfiltration analysis. **(A)** Box diagram displaying differential analysis results for 22 types of immune cells between the disease and control groups; **(B)** Correlation analysis between the hub genes and immune cells, as well as between different immune cells *P<0.05, **P<0.01, ***P<0.001. ns, no significance.

We performed an immunoinfiltration analysis on the datasets and presented the differences in 22 types of immune cells between the disease and the control groups (**Figure 4A**), revealing significant differences in plasma cells, NK cells activated, macrophages M0, macrophages M1, macrophages M2, dendritic cells activated, mast cells resting, mast cells activated and neutrophils between groups. Meanwhile, we analyzed the correlation between the hub genes and immune cells, as well as between different immune cells (**Figure 4B**). The results showed that both Ube2d3 and Dlat were significantly positively correlated with neutrophils.

### 3.5 Single-cell analysis

**Figure 5:**
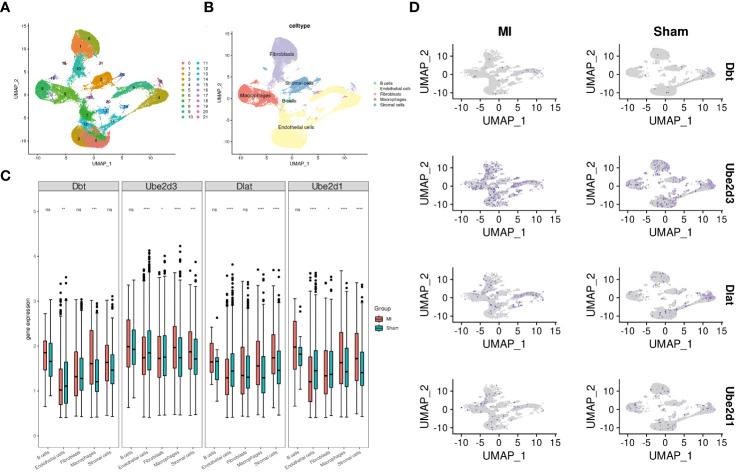
Single-cell analysis. **(A)** UMAP diagram illustrating cell clustering; **(B)** UMAP diagram displaying cell annotation; **(C)** Boxplots presenting the expression patterns of hub genes across individual cells; **(D)** UMAP diagram demonstrating the distribution of hub genes in cells.

We performed single-cell analysis on the GSE214611 dataset. Quality control procedures were applied to the cells within the dataset (**Supplementary Figures 3A, B**). All the cells were subsequently classified into 21 cell clusters (**Figure 5A**), and subjected to cell annotation (**Figure 5B**), resulting in the identification of five major cell types: B cells, Endothelial cells, Fibroblasts, Macrophages, and Stromal cells. Notably, Ube2d3 showed significant differences in Endothelial cells, Fibroblasts, Macrophages, and Stromal cells (**Figure 5C**), and it displayed the most widespread distribution among these cell types (**Figure 5D**).

### 3.6 Ube2d3 is highly expressed in MI model mice

**Figure 6:**
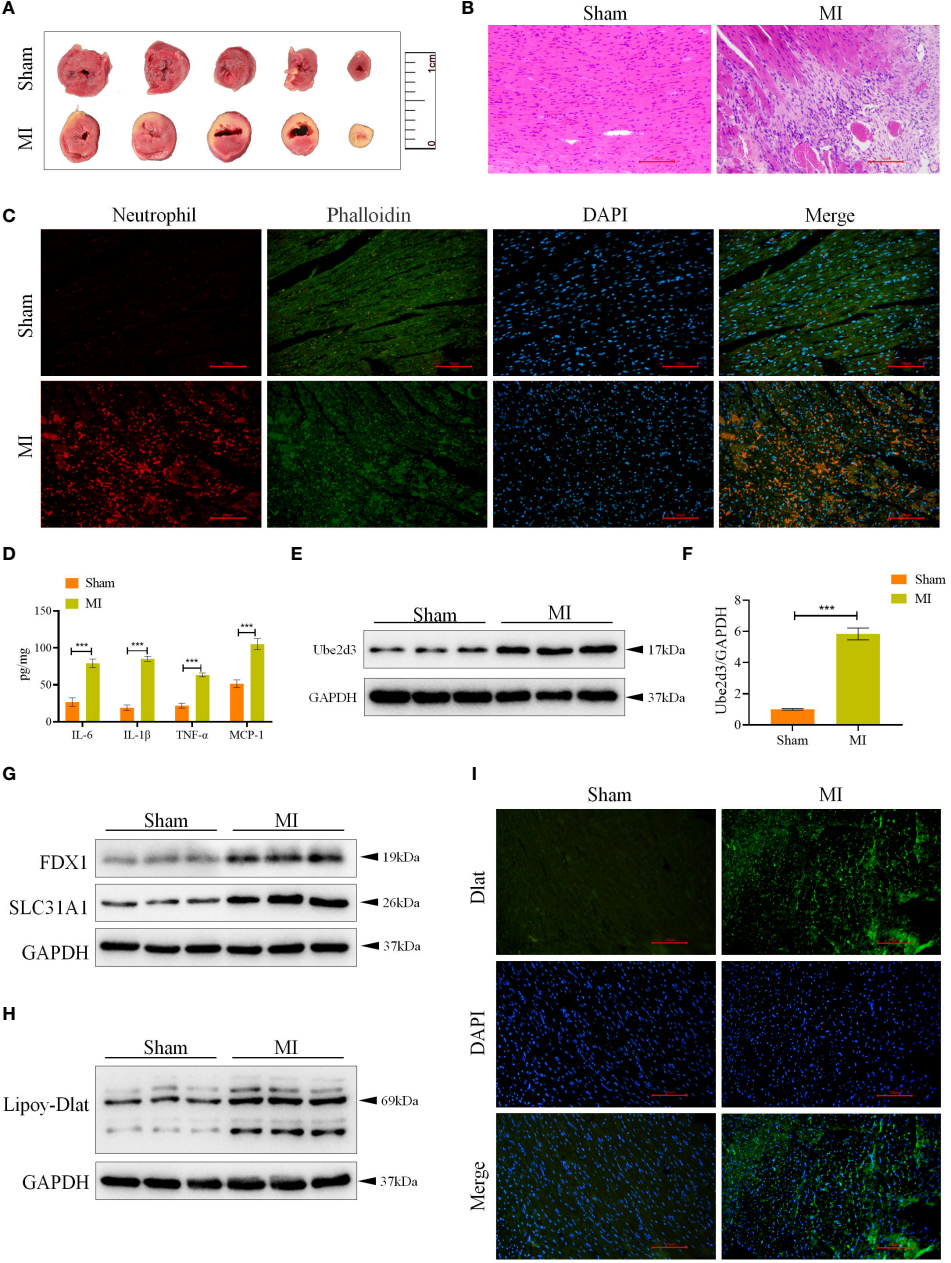
Ube2d3 is highly expressed in MI model mice. **(A)** TTC staining revealing myocardial tissue infarction, with normal myocardial tissue displaying red, and infarcted areas displaying pale color. (n=3) **(B)** HE staining demonstrating myocardial tissue damage in the MI group, characterized by obvious myocardial cell necrosis, edema and disordered arrangement. Scale bar: 100 μm, respectively. (n=6); **(C)** Immunofluorescence staining showing neutrophil infiltration in myocardial tissue. Neutrophils were labeled using neutrophil (red), myocardial cytoskeleton was labeled using phalloidin (green), and nuclei were stained with DAPI. Scale bar: 100 μm, respectively. (n=6) **(D)** ELISA assay revealing the levels of inflammatory factors in myocardial tissue. (n=6, ***P<0.001) **(E)** WB detection of Ube2d3 expression in myocardial tissue. (n=6); **(F)** qRT-PCR detection of Ube2d3 expression in myocardial tissue. (n=6, ***P<0.001) **(G)** WB detection of the expression of FDX1 and SLC31A1 in myocardial tissue. (n=6) **(H)** WB detection of the lipoylation level of Dlat in myocardial tissue. (n=6) **(I)** Immunofluorescence staining illustrating Dlat oligomerization in myocardial tissue. Scale bar: 100 μm, respectively. (n=6). Statistical data in this figure are presented as mean values ± SD. Statistical significance was determined by Two-tailed t test.

Based on the results of single-cell analysis as well as relevant literature (10), we selected the CRG Ube2d3 for follow-up studies. To elucidate the mechanism of Ube2d3 in MI, we constructed a mouse model of MI. Through TTC staining, we observed relatively obvious white infarct area in the heart tissue of mice in the MI group (**Figure 6A**). Histological examination via HE staining revealed evident myocardial cell necrosis, edema, neutrophil infiltration, and arrangement disorder in the myocardial tissue of mice in the MI group, whereas mice the Sham group displayed neatly arranged fibers with only a minimal degree of neutrophil infiltration within the myocardial tissue (**Figure 6B**). Furthermore, according to immunofluorescence results, substantial neutrophil infiltration was observed in the myocardial tissue of mice in the MI group (**Figure 6C**). Besides, we also measured the expression of inflammatory factors, including IL-6, IL-1β, TNF-α and MCP-1, in the myocardial tissue. The results indicated a significant elevation of these inflammatory factors in the MI group (**Figure 6D**), signifying a state of immune activation in the myocardial tissue. Through qRT-PCR and WB detection, we found a notable upregulation in the expression of Ube2d3 in the myocardial tissue in the MI group (**Figures 6E, F**), which was consistent with the results of our bioinformatics analysis. Additionally, the expression of cuproptosis-related proteins, FDX1 and SLC31A1, as well as the lipoylation level of Dlat in the myocardial tissue of mice was found to be significantly increased in the MI group (**Figures 6G, H**). Meanwhile, we also observed a significant aggregation of Dlat (**Figure 6I**). The above results collectively suggest that cuproptosis may indeed play an important role in the progression of MI.

### 3.7 Ube2d3 is highly expressed in OGD-treated AC16 cells

**Figure 7:**
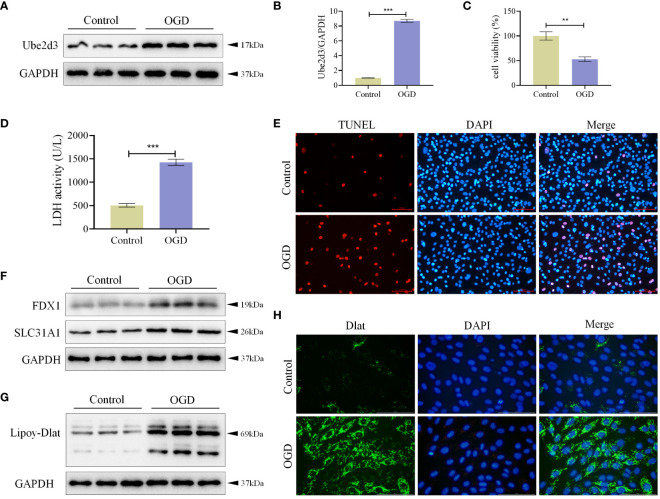
Ube2d3 is highly expressed in OGD-treated AC16 cells. **(A)** WB detection of Ube2d3 expression in cardiomyocytes AC16. (n=3) **(B)** qRT-PCR detection of Ube2d3 expression in cardiomyocytes AC16. (n=3, ***P<0.001) **(C)** MTT detection of cell activity. (n=3, **P<0.01) **(D)** Quantification of cytotoxicity by measuring the activity of LDH released into the culture medium from cells with ruptured plasma membranes. (n=3, ***P<0.001) **(E)** TUNEL staining for cell death detection, with red fluorescence indicating apoptotic cells. Under the action of TdT Enzyme, fluorescein labeled dUTP can be linked to the 3 ‘-OH end of broken DNA in apoptotic cells, showing red fluorescence. Scale bar: 100 μm, respectively. (n=3) **(F)** WB detection of the expression of FDX1 and SLC31A1 in cardiomyocytes AC16. (n=3) **(G)** WB detection of the lipoylation level of Dlat in cardiomyocytes AC16. (n=3) **(H)** Immunofluorescence staining showing Dlat oligomerization in cardiomyocytes. Scale bar: 100 μm, respectively. (n=3) Statistical data in this figure are presented as mean values ± SD. Statistical significance was determined by Two-tailed t test.

We treated AC16 cells with OGD, and measured Ube2d3 expression levels via qRT-PCR and WB detection. As a results, Ube2d3 expression was revealed to be significantly elevated in the OGD group (**Figures 7A, B**), which aligned with our bioinformatics analysis results. Concomitantly, we observed a significant reduction in cell viability in the OGD group (**Figure 7C**) and a remarkable increase in cell death rates (**Figures 7D, E**) based on relevant test results. Additionally, the expressions of FDX1 and SLC31A1, as well as the lipoylation level of Dlat were significantly increased in the OGD group (**Figures 7F, G**). Meanwhile, we also observed a significant aggregation of Dlat (**Figure 7H**). These results suggest that during MI development, Ube2d3 is closely related to cuproptosis and can aggravate hypoxic injury of cardiomyocytes.

### 3.8 Ube2d3 promotes hypoxic damage of cardiomyocytes AC16 by regulating cuproptosis

**Figure 8:**
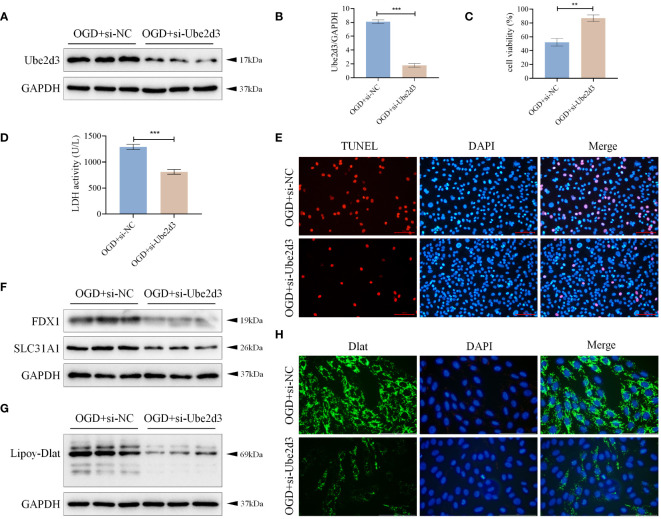
Ube2d3 promotes hypoxic damage of cardiomyocytes AC16 by regulating cuproptosis. **(A)** WB detection of Ube2d3 expression in cardiomyocytes AC16. (n=3) **(B)** qRT-PCR detection of Ube2d3 expression in cardiomyocytes AC16. (n=3, ***P<0.001) **(C)** MTT detection of cell activity. (n=3, **P<0.01) **(D)** Quantification of cytotoxicity by measuring the activity of LDH released into the culture medium from cells with ruptured plasma membranes. (n=3, ***P<0.001) **(E)** TUNEL staining for cell death detection, with red fluorescence indicating apoptotic cells. Under the action of TdT Enzyme, fluorescein labeled dUTP can be linked to the 3 ‘-OH end of broken DNA in apoptotic cells, showing red fluorescence. Scale bar: 100 μm, respectively. (n=3) **(F)** WB detection of the expression of FDX1 and SLC31A1 in cardiomyocytes AC16. (n=3) **(G)** WB detection of the lipoylation level of Dlat in cardiomyocytes AC16. (n=3) **(H)** Immunofluorescence staining showing Dlat oligomerization in cardiomyocytes. Scale bar: 100 μm, respectively. (n=3) Statistical data in this figure are presented as mean values ± SD. Statistical significance was determined by Two-tailed t test.

To further investigate the mechanism of action of Ube2d3, we knocked down the expression of Ube2d3 in OGD-treated AC16 cells (**Figures 8A, B**), and found increased cell activity (**Figure 8C**), significantly reduced number of cell death (**Figures 8D, E**), markedly lowered expressions of FDX1 and SLC31A1, as well as obviously decreased lipoylation level of Dlat (**Figures 8F, G**) in the OGD+si-Ube2d3 group when compared with the OGD+si-NC group. Moreover, Dlat did not display significant aggregation (**Figure 8H**) in the OGD+si-Ube2d3 group. Therefore, we infer that Ube2d3 can promote hypoxic damage in cardiomyocytes AC16 by regulating cuproptosis.

## 4 Discussion

Cuproptosis is a newly recognized form of cell death first proposed in 2022. This process primarily involves the targeted accumulation of Cu^2+^ within mitochondria. Cu^2+^ binds to lipoylated proteins engaged in the tricarboxylic acid (TCA) cycle of mitochondrial respiration (11). This interaction triggers the oligomerization of lipoylated proteins, subsequently resulting in the downregulation of Fe-S cluster protein expression (12–14), which in turn induces protein toxic stress and ultimately leads to cell death (5). Notably, FDX1 plays a multifaceted role in this mechanism. It not only participates in the formation of lipoylation of Dlat, but also aids in the conversion of Cu^2+^ to toxic Cu^+^, thereby inhibiting the synthesis of Fe-S cluster proteins. The level of Cu^2+^ has been identified as a critical factor in the development of cardiovascular diseases, including myocardial ischemia/reperfusion injury, heart failure, atherosclerosis, and arrhythmias (15). Importantly, it’s worth emphasizing that the investigation of cuproptosis as a potential intervention target is still in its infancy within the realm of MI research. In this study, by performing bioinformatics analysis, we screened out CRGs, Dbt, Dlat, Ube2d1 and Ube2d3, all of which may hold significant roles in the progression of MI.

According to relevant literature, Dbt can regulate the Hippo signaling pathway to affect the tumor growth of kidney renal clear cell carcinoma (16); DLAT has been identified as a key factor in the process of cuproptosis (11); Ube2D1 has been linked to the malignant progression of gastric cancer and breast cancer (17, 18); Ube2d3 has been reported to promote p62 ubiquitination and aggravate impaired autophagic flow in myocardial ischemia-reperfusion injury (10). However, there has been no study investigating whether Ube2d3 can affect MI by inducing cuproptosis. In summary, we select Ube2d3 for subsequent experimental verification.

Based on our investigations at the animal level, we found significant changes in FDX1, SLC31A1 (a protein that regulates Cu^2+^ concentration) (19), and the lipoylation levels of Dlat within the myocardial tissues of MI model mice. Moreover, Dlat aggregation was also observed. From these findings, we infer that cuproptosis may affect the development of MI. Subsequently, upon knocking down the expression of Ube2d3 in OGD-treated AC16 cells, we noted significant changes in the levels of FDX1, SLC31A1, and the lipoylation of Dlat within cardiomyocytes. Consequently, we hypothesize that Ube2d3 may induce cuproptosis to affect the development of MI. Mengdan Miao et al. have confirmed the role of six cuproptosis- and ferroptosis-related genes (CXCL2, DDIT3, DUSP1, CDKN1A, TLR4 and STAT3) in predicting the progression of AMI (20). Additionally, Zheng Liu et al. identified CRG GLS as a diagnostic gene for AMI (21). The experimental results of this study align with existing literature, reinforcing each other and establishing a crucial foundation for understanding the role of cuproptosis in the prevention and treatment of MI. Cuproptosis also plays an important role in the pathogenesis of vascular diseases (15), including atherosclerosis, stroke, ischemia-reperfusion injury and heart failure (22). Shengqi Huo et al. demonstrated that AGEs in diabetic cardiomyopathy can promote cuproptosis through the ATF3/SPI1/SLC31A1 signaling pathway (13). As a new research focus, cuproptosis has raised the attention of many scholars and brought a series of breakthroughs, offering promising insights for addressing diverse diseases. Though existing research on the mechanisms of cuproptosis remains relatively limited, there is compelling evidence to suggest that cuproptosis plays an important role in cancer progression. For instance, cuproptosis affects the activity of immune cells in the tumor microenvironment of kidney renal clear cell carcinoma (23); Dexmedetomidine mitigates cerebral infarction in I/R rat models by blocking FDX1-mediated cuproptosis (24); Cuproptosis has been shown to impact both presynaptic and postsynaptic regulatory mechanisms in a mouse model of cognitive dysfunction (25). Drawing from existing literature, it is believed that continued in-depth investigation of cuproptosis in various diseases and a deeper analysis of its molecular mechanisms will enhance our comprehension of cuproptosis in the field of MI.

In the acute stage of MI, the myocardium showed massive and flaky coagulated necrosis, hyperemia and edema, accompanied by significant inflammatory cell infiltration. To elucidate the relationship between the key genes and immune cells, we conducted immune infiltration analysis. The results revealed notable differences in NK cells, dendritic cells, mast cells and other immune cells between the control group and the disease group. Specifically, Ube2d3 exhibited a significant positive correlation with neutrophils. NK cells, dendritic cells and mast cells are of critical importance in promoting angiogenesis and angiogenesis and inhibiting myocardial fibrosis after MI (26–28). Tianxiao Liu et al. found that group 2 innate lymphoid cells indirectly contribute to cardiac function repair after MI through dendritic cells (29). Additionally, atorvastatin has been shown to inhibit TLR-4/NF-κB activation after MI by inducing tolerogenic dendritic cells to improve myocardial remodeling (30). Furthermore, exercise has been found to enhance fibrosis and cardiac function in MI rats by inhibiting mast cell-released trypsin (31). The relationship between Ube2d3 and immune cells is relatively understudied, but existing knowledge indicates that Ube2d3 activates the NF-κB pathway to promote chemokine production and myeloid cell invasion in tumors (32). Our current study suggests that the interaction between Ube2d3 and neutrophils may also play an important regulatory role in MI progression.

Here, it should be acknowledged that this study has certain limitations. In clinical practice, the circumstances of MI patients tend to be rather intricate. MI occurrence is related to a variety of factors such as age and lifestyle, and individual differences also exist. Consequently, it remains uncertain whether our findings can have a significant impact on the clinical treatment of MI and the delay of MI progression. It is expected that this study can serve as a foundational step towards elucidating the potential molecular mechanisms involved in MI prevention and treatment. Additionally, we aim to provide fresh perspectives and insights for the targeted treatment of MI based on cuproptosis. In the future, further elucidation of the regulatory mechanism of Ube2d3 in cuproptosis is warranted. Concurrently, it is also imperative to apply our research findings in clinical practice, with the hope of developing a specific drug targeting Ube2d3 for MI.

In conclusion, with the aid of bioinformatics techniques, Ube2d3 was screened out and identified as key CRGs that affects the development of MI. Moreover, Ube2d3 was also confirmed to induce cuproptosis, leading to cardiomyocyte death, consequently affecting the progression of MI.

## Data availability statement

The datasets presented in this study can be found in online repositories. The names of the repository/repositories and accession number(s) can be found in the article/**Supplementary Material**.

## Ethics statement

The animal study was approved by the Institutional Animal Care and Use Committee of Zhejiang Center of Laboratory Animals. The study was conducted in accordance with the local legislation and institutional requirements.

## Author contributions

MY: Conceptualization, Data curation, Project administration, Writing – original draft. YW: Formal analysis, Methodology, Writing – original draft. LH: Formal analysis, Investigation, Methodology, Software, Writing – original draft. XS: Data curation, Formal analysis, Investigation, Methodology, Supervision, Writing – original draft. SH: Project administration, Resources, Writing – review & editing.

## Supplementary material

Supplementary Figure 1 Integration of the three datasets. **(A)** PCA plots illustrating the aggregation of samples in the three datasets before data merging; **(B)** PCA plots illustrating the aggregation of samples in the three datasets after data merging; **(C)** Box plot showing the expression range of each sample after data merging without normalization; **(D)** Box plot showing the expression range of each sample after data merging and standardization.

Supplementary Figure 2 ssGSEA. **(A)** ssGSEA of Dlat; **(B)** ssGSEA of Ube2d1; **(C)** ssGSEA of Ube2d3; **(D)** ssGSEA of Dbt.

Supplementary Figure 3 Single-cell analysis. **(A)** Violin plot illustrating the amounts of nFeature RNA, nCount RNA, and percent. mt for each sample in the single-cell dataset before data screening; **(B)** Violin plot illustrating the amounts of nFeature RNA, nCount RNA, and percent. mt for each sample in the single-cell dataset after data filtering.

## References

[1] Li J Wu F Li C Sun S Feng C Wu H. The cuproptosis-related signature predicts prognosis and indicates immune microenvironment in breast cancer. Front Genet (2022) 13:977322. doi: 10.3389/fgene.2022.977322. 36226193 PMC9548612

[2] Anderson JL Morrow DA. Acute myocardial infarction. N Engl J Med (2017) 376:2053–64. doi: 10.1056/NEJMra1606915. 28538121

[3] Sagris M Antonopoulos AS Theofilis P Oikonomou E Siasos G Tsalamandris S. Risk factors profile of young and older patients with myocardial infarction. Cardiovasc Res (2022) 118:2281–92. doi: 10.1093/cvr/cvab264. 34358302

[4] Saito Y Oyama K Tsujita K Yasuda S Kobayashi Y. Treatment strategies of acute myocardial infarction: updates on revascularization, pharmacological therapy, and beyond. J Cardiol (2023) 81:168–78. doi: 10.1016/j.jjcc.2022.07.003. 35882613

[5] Tsvetkov P Coy S Petrova B Dreishpoon M Verma A Abdusamad M. Copper induces cell death by targeting lipoylated TCA cycle proteins. Science (2022) 375:1254–61. doi: 10.1126/science.abf0529. PMC9273333 35298263

[6] Lim SY Dayal H Seah SJ Tan RPW Low ZE Laserna AKC. Plasma metallomics reveals potential biomarkers and insights into the ambivalent associations of elements with acute myocardial infarction. J Trace Elem Med Biol (2023) 77:127148. doi: 10.1016/j.jtemb.2023.127148. 36905853

[7] Chen L Min J Wang F. Copper homeostasis and cuproptosis in health and disease. Signal Transduct Target Ther (2022) 7:378. doi: 10.1038/s41392-022-01229-y. 36414625 PMC9681860

[8] Liu H. Pan-cancer profiles of the cuproptosis gene set. Am J Cancer Res (2022) 12:4074–81. doi: 10.3389/fonc.2022.952290. PMC9442004 36119826

[9] Zhang G Sun J Zhang X. A novel Cuproptosis-related LncRNA signature to predict prognosis in hepatocellular carcinoma. Sci Rep (2022) 12:11325. doi: 10.1038/s41598-022-15251-1. 35790864 PMC9256635

[10] Wang X Yang P Jiang Y Xu Y Wang N Rao P. UBE2D3 contributes to myocardial ischemia-reperfusion injury by regulating autophagy in dependence of p62/SQSTM1. Cell Signal (2021) 87:110118. doi: 10.1016/j.cellsig.2021.110118. 34391873

[11] Ke C Dai S Xu F Yuan J Fan S Chen Y. Cuproptosis regulatory genes greatly contribute to clinical assessments of hepatocellular carcinoma. BMC Cancer (2023) 23:25. doi: 10.1186/s12885-022-10461-2. 36611155 PMC9824945

[12] Li X Dai Z Liu J Sun Z Li N Jiao G. Characterization of the functional effects of ferredoxin 1 as a cuproptosis biomarker in cancer. Front Genet (2022) 13:969856. doi: 10.3389/fgene.2022.969856. 36226187 PMC9549589

[13] Huo S Wang Q Shi W Peng L Jiang Y Zhu M. ATF3/SPI1/SLC31A1 signaling promotes cuproptosis induced by advanced glycosylation end products in diabetic myocardial injury. Int J Mol Sci (2023) 24:1667. doi: 10.3390/ijms24021667. PMC9862315 36675183

[14] Dreishpoon MB Bick NR Petrova B Warui DM Cameron A Booker SJ. FDX1 regulates cellular protein lipoylation through direct binding to LIAS. J Biol Chem (2023) 299:105046. doi: 10.1016/j.jbc.2023.105046. 37453661 PMC10462841

[15] Wang D Tian Z Zhang P Zhen L Meng Q Sun B. The molecular mechanisms of cuproptosis and its relevance to cardiovascular disease. BioMed Pharmacother (2023) 163:114830. doi: 10.1016/j.biopha.2023.114830. 37150036

[16] Miao D Wang Q Shi J Lv Q Tan D Zhao C. N6-methyladenosine-modified DBT alleviates lipid accumulation and inhibits tumor progression in clear cell renal cell carcinoma through the ANXA2/YAP axis-regulated Hippo pathway. Cancer Commun (Lond) (2023) 43:480–502. doi: 10.1002/cac2.12413. 36860124 PMC10091108

[17] Xie H He Y Wu Y Lu Q. Silencing of UBE2D1 inhibited cell migration in gastric cancer, decreasing ubiquitination of SMAD4. Infect Agent Cancer (2021) 16:63. doi: 10.1186/s13027-021-00402-2. 34743754 PMC8574036

[18] Guan XQ Yuan XN Feng KX Shao YC Liu Q Yang ZL. IGF2BP2-modified UBE2D1 interacts with Smad2/3 to promote the progression of breast cancer. Am J Cancer Res (2023) 13:2948–68. PMC10408479 37560007

[19] Prasad Panda S Kesharwani A. Micronutrients/miRs/ATP networking in mitochondria: Clinical intervention with ferroptosis, cuproptosis, and calcium burden. Mitochondrion (2023) 71:1–16. doi: 10.1016/j.mito.2023.05.003. 37172668

[20] Miao M Cao S Tian Y Liu D Chen L Chai Q. Potential diagnostic biomarkers: 6 cuproptosis- and ferroptosis-related genes linking immune infiltration in acute myocardial infarction. Genes Immun (2023) 24:159–70. doi: 10.1038/s41435-023-00209-8. PMC10435388 37422588

[21] Liu Z Wang L Xing Q Liu X Hu Y Li W. Identification of GLS as a cuproptosis-related diagnosis gene in acute myocardial infarction. Front Cardiovasc Med (2022) 9:1016081. doi: 10.3389/fcvm.2022.1016081. 36440046 PMC9691691

[22] Chen X Cai Q Liang R Zhang D Liu X Zhang M. Copper homeostasis and copper-induced cell death in the pathogenesis of cardiovascular disease and therapeutic strategies. Cell Death Dis (2023) 14:105. doi: 10.1038/s41419-023-05639-w. 36774340 PMC9922317

[23] Xie M Cheng B Yu S He Y Cao Y Zhou T. Cuproptosis-related MiR-21-5p/FDX1 axis in clear cell renal cell carcinoma and its potential impact on tumor microenvironment. Cells (2022) 12:173. doi: 10.3390/cells12010173. PMC9818076 36611966

[24] Guo Q Ma M Yu H Han Y Zhang D. Dexmedetomidine enables copper homeostasis in cerebral ischemia/reperfusion via ferredoxin 1. Ann Med (2023) 55:2209735. doi: 10.1080/07853890.2023.2209735. 37162502 PMC10173798

[25] Zhang Y Zhou Q Lu L Su Y Shi W Zhang H. Copper induces cognitive impairment in mice via modulation of cuproptosis and CREB signaling. Nutrients (2023) 15:972. doi: 10.3390/nu15040972. PMC9958748 36839332

[26] Sun K Li YY Jin J. A double-edged sword of immuno-microenvironment in cardiac homeostasis and injury repair. Signal Transduct Target Ther (2021) 6:79. doi: 10.1038/s41392-020-00455-6. 33612829 PMC7897720

[27] Varricchi G Marone G Kovanen PT. Cardiac mast cells: underappreciated immune cells in cardiovascular homeostasis and disease. Trends Immunol (2020) 41:734–46. doi: 10.1016/j.it.2020.06.006. 32605802

[28] Haider N Bosca L Zandbergen HR Kovacic JC Narula N Gonzalez-Ramos S. Transition of macrophages to fibroblast-like cells in healing myocardial infarction. J Am Coll Cardiol (2019) 74:3124–35. doi: 10.1016/j.jacc.2019.10.036. PMC7425814 31856969

[29] Liu T Meng Z Liu J Li J Zhang Y Deng Z. Group 2 innate lymphoid cells protect mouse heart from myocardial infarction injury via interleukin 5, eosinophils, and dendritic cells. Cardiovasc Res (2023) 119:1046–61. doi: 10.1093/cvr/cvac144. PMC10153644 36063432

[30] Wang Q Chen Z Guo J Peng X Zheng Z Chen H. Atorvastatin-induced tolerogenic dendritic cells improve cardiac remodeling by suppressing TLR-4/NF-κB activation after myocardial infarction. Inflammation Res (2023) 72:13–25. doi: 10.1007/s00011-022-01654-3. 36315279

[31] Bayat M Chien S Chehelcheraghi F. Aerobic exercise-assisted cardiac regeneration by inhibiting tryptase release in mast cells after myocardial infarction. BioMed Res Int (2021) 2021:5521564. doi: 10.1155/2021/5521564. 34212030 PMC8205576

[32] Obacz J Archambeau J Lafont E Nivet M Martin S Aubry M. IRE1 endoribonuclease signaling promotes myeloid cell infiltration in glioblastoma. Neuro Oncol (2023) 28:noad256. doi: 10.1093/neuonc/noad256. PMC11066906 38153426

