# Supplementary figures and images for: Comprehensive bioinformatics analysis reveals the role of cuproptosis-related gene Ube2d3 in myocardial infarction
Source: Front Immunol. 2024 Feb 19;15:1353111. doi: 10.3389/fimmu.2024.1353111 (PMC10909922; doi:10.3389/fimmu.2024.1353111)

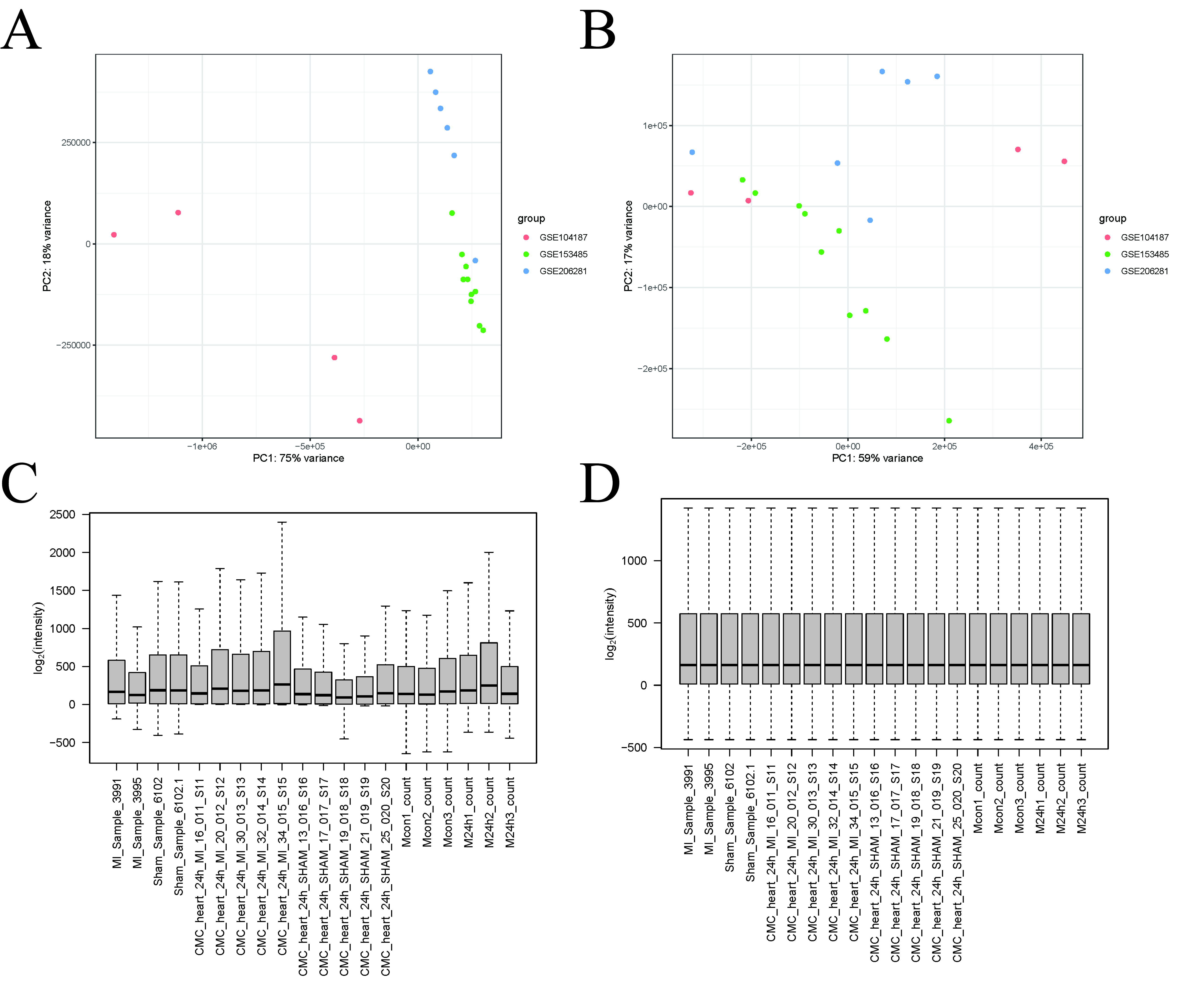

Supplement: Supplementary Figure 1 — Integration of the three datasets. (A) PCA plots illustrating the aggregation of samples in the three datasets before data merging; (B) PCA plots illustrating the aggregation of samples in the three datasets after data merging; (C) Box plot showing the expression range of each sample after data merging without normalization; (D) Box plot showing the expression range of each sample after data merging and standardization. [file Image_1.jpeg]

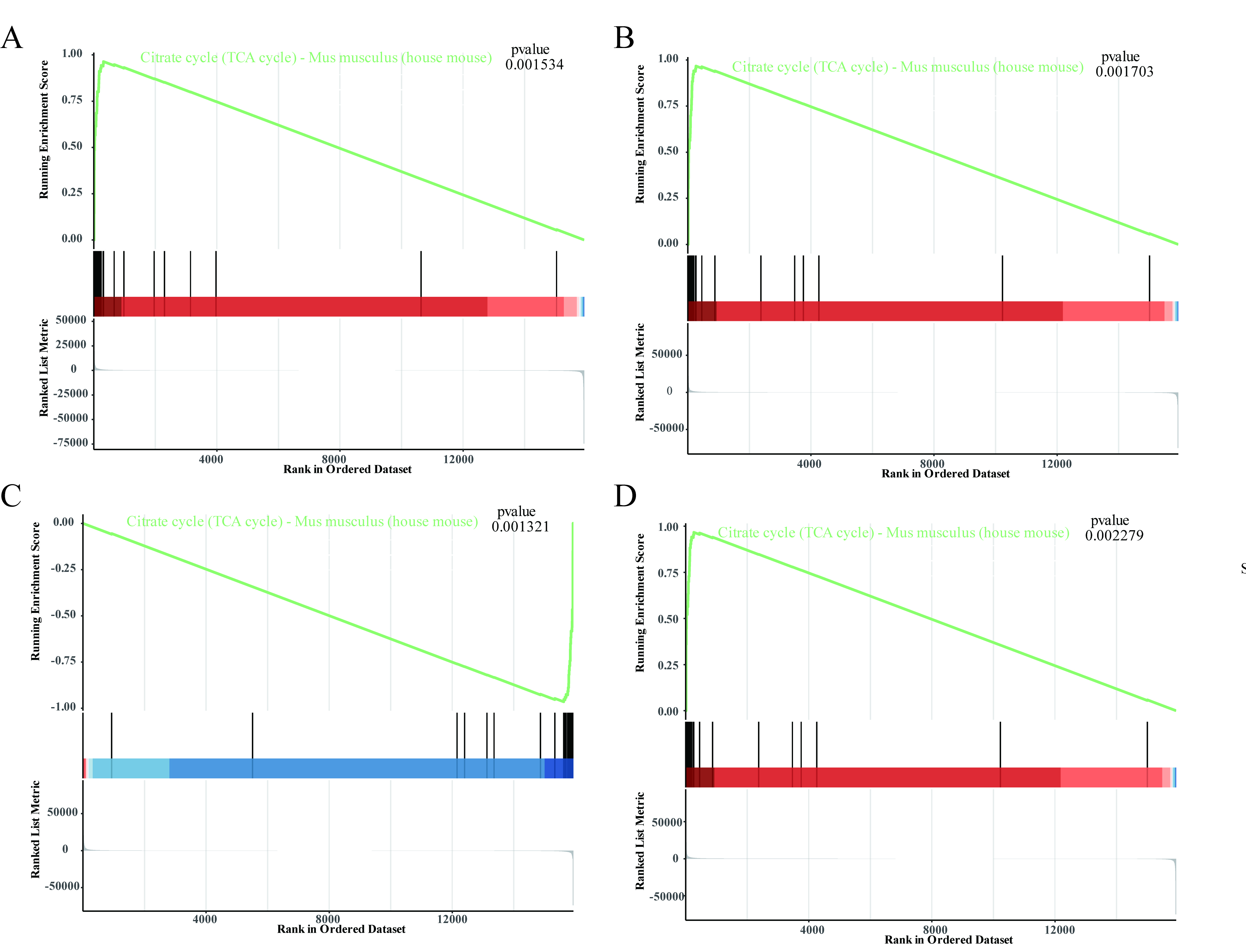

Supplement: Supplementary Figure 2 — ssGSEA. (A) ssGSEA of Dlat; (B) ssGSEA of Ube2d1; (C) ssGSEA of Ube2d3; (D) ssGSEA of Dbt. [file Image_2.jpeg]

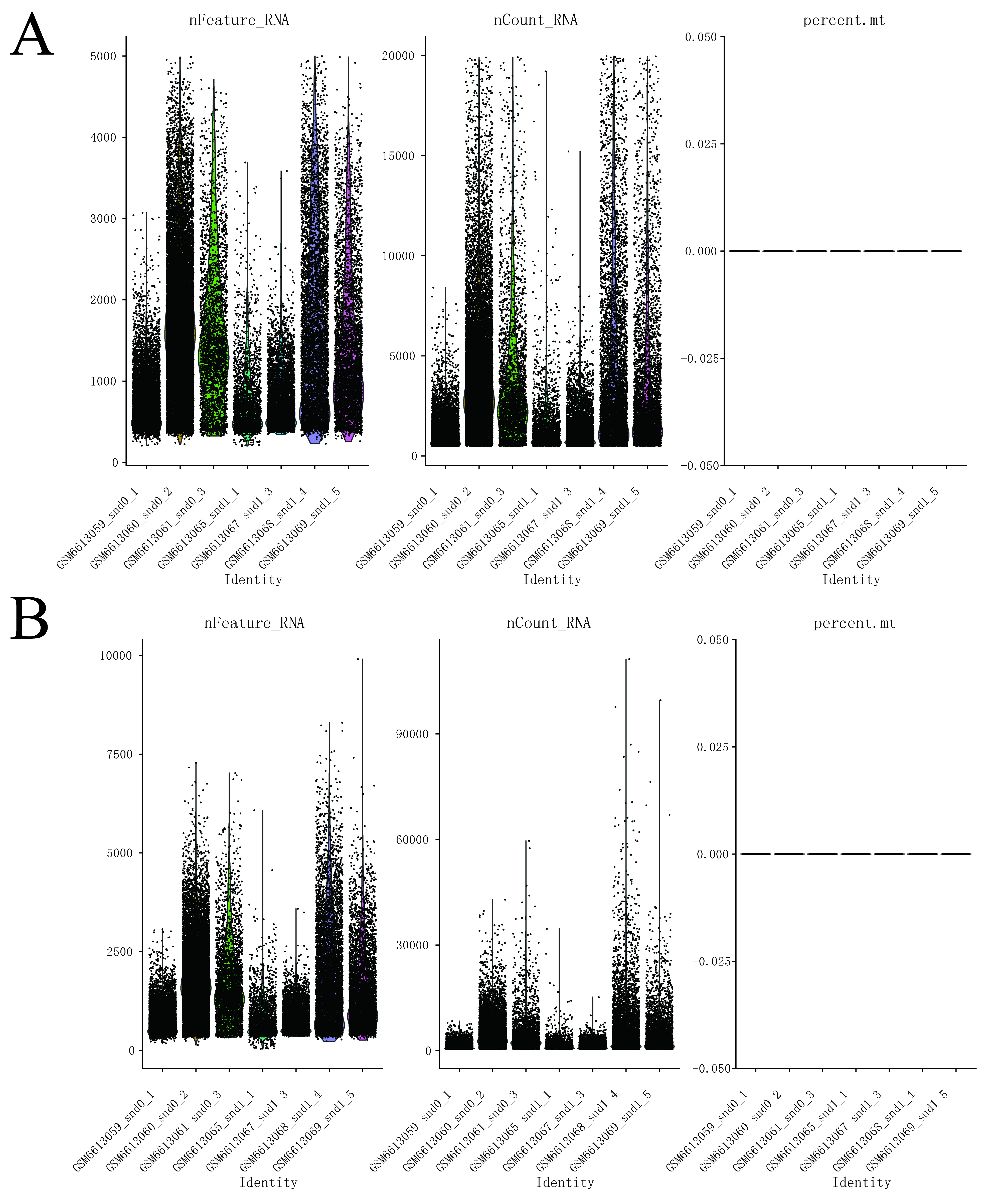

Supplement: Supplementary Figure 3 — Single-cell analysis. (A) Violin plot illustrating the amounts of nFeature RNA, nCount RNA, and percent. mt for each sample in the single-cell dataset before data screening; (B) Violin plot illustrating the amounts of nFeature RNA, nCount RNA, and percent. mt for each sample in the single-cell dataset after data filtering. [file Image_3.jpeg]
