# Supplementary material for: Comprehensive bioinformatics analysis reveals the role of cuproptosis-related gene Ube2d3 in myocardial infarction
Source: Front Immunol. 2024 Feb 19;15:1353111. doi: 10.3389/fimmu.2024.1353111 (PMC10909922; doi:10.3389/fimmu.2024.1353111)
Supplement: Supplementary file 4 [file Table_1.docx]

Supplementary table 1 **cuproptosis genes**

| **EntrezID** | **Gene Name** | **Symbol** |
| --- | --- | --- |
| 1738 | dihydrolipoamide dehydrogenase | DLD |
| 5162 | pyruvate dehydrogenase E1 subunit beta | PDHB |
| 540 | ATPase copper transporting beta | ATP7B |
| 538 | ATPase copper transporting alpha | ATP7A |
| 1737 | dihydrolipoamide S-acetyltransferase | DLAT |
| 1743 | dihydrolipoamide S-succinyltransferase | DLST |
| 1317 | solute carrier family 31 member 1 | SLC31A1 |
| 1629 | dihydrolipoamide branched chain transacylase E2 | DBT |
| 2230 | ferredoxin 1 | FDX1 |
| 11019 | lipoic acid synthetase | LIAS |
| 2653 | glycine cleavage system protein H | GCSH |
| 5160 | pyruvate dehydrogenase E1 subunit alpha 1 | PDHA1 |
| 51601 | lipoyltransferase 1 | LIPT1 |
| 4520 | metal regulatory transcription factor 1 | MTF1 |
| 2744 | glutaminase | GLS |
| 1029 | cyclin dependent kinase inhibitor 2A | CDKN2A |
| 7322 | ubiquitin conjugating enzyme E2 D2 | UBE2D2 |
| 388753 | cytochrome c oxidase assembly factor 6 | COA6 |
| 10063 | cytochrome c oxidase copper chaperone COX17 | COX17 |
| 51619 | ubiquitin conjugating enzyme E2 D4 (putative) | UBE2D4 |
| 7321 | ubiquitin conjugating enzyme E2 D1 | UBE2D1 |
| 8350 | H3 clustered histone 1 | H3C1 |
| 29126 | CD274 molecule | CD274 |
| 5604 | mitogen-activated protein kinase kinase 1 | MAP2K1 |
| 5163 | pyruvate dehydrogenase kinase 1 | PDK1 |
| 6341 | synthesis of cytochrome C oxidase 1 | SCO1 |
| 7323 | ubiquitin conjugating enzyme E2 D3 | UBE2D3 |
| 6647 | superoxide dismutase 1 | SOD1 |
| 5250 | solute carrier family 25 member 3 | SLC25A3 |
| 9706 | unc-51 like autophagy activating kinase 2 | ULK2 |
| 1356 | ceruloplasmin | CP |
| 8639 | amine oxidase copper containing 3 | AOC3 |
| 1318 | solute carrier family 31 member 2 | SLC31A2 |
| 1621 | dopamine beta-hydroxylase | DBH |
| 8408 | unc-51 like autophagy activating kinase 1 | ULK1 |
| 7422 | vascular endothelial growth factor A | VEGFA |
| 9973 | copper chaperone for superoxide dismutase | CCS |
| 5605 | mitogen-activated protein kinase kinase 2 | MAP2K2 |
| 475 | antioxidant 1 copper chaperone | ATOX1 |
| 107075310 | MT-CO2 pseudogene 12 | MTCO2P12 |
| 7299 | tyrosinase | TYR |
| 114548 | NLR family pyrin domain containing 3 | NLRP3 |
| 4780 | NFE2 like bZIP transcription factor 2 | NFE2L2 |
|  |  | C0X11 |
|  |  | PDE38 |
|  |  | LOX12 |
|  |  | ATP78 |
|  |  | ATPTA |
|  |  | LIPIT1 |
